# Illness cognition as a predictor of exercise habits and participation in cardiac prevention and rehabilitation programs after acute coronary syndrome

**DOI:** 10.1186/1471-2458-13-956

**Published:** 2013-10-12

**Authors:** Orna Reges, Noa Vilchinsky, Morton Leibowitz, Abdulrahem Khaskia, Morris Mosseri, Jeremy D Kark

**Affiliations:** 1 Hebrew University-Hadassah School of Public Health and Community Medicine, Ein Kerem, Jerusalem, Israel; 2 Clalit Research Institute, Tel-Aviv, Israel; 3 Department of Cardiology, Meir Medical Center, Kfar-Saba, Israel; 4 Department of Psychology, Bar-Ilan University, Ramat-Gan, Israel; 5 Department of Cardiology, N.Y.U. School of Medicine, New York, USA

## Abstract

**Background:**

Despite well-established medical recommendations, many cardiac patients do not exercise regularly either independently or through formal cardiac prevention and rehabilitation programs (CPRP). This non-adherence is even more pronounced among minority ethnic groups. Illness cognition (IC), i.e. the way people *perceive* the situation they encounter, has been recognized as a crucial determinant of health-promoting behavior. Few studies have applied a cognitive perspective to explain the disparity in exercising and CPRP attendance between cardiac patients from different ethnic backgrounds. Based on the Health Belief Model (HBM) and the Common Sense Model (CSM), the objective was to assess the association of IC with exercising and with participation in CPRP among Jewish/majority and Arab/minority patients hospitalized with acute coronary syndrome.

**Methods:**

Patients (N = 420) were interviewed during hospitalization (January-2009 until August- 2010) about IC, with 6-month follow-up interviews about exercise habits and participation in CPRP. Determinants that predict active lifestyle and participation in CPRP were assessed using backward stepwise logistic regression.

**Results:**

Perceived susceptibility to heart disease and sense and personal control were independently associated with exercising 6 months after the acute event (OR = 0.58, 95% CI: 0.42-0.80 and OR = 1.09, 95% CI: 1.02-1.17, per unit on a 5-point scale). Perceived benefits of regular exercise and a sense of personal control were independently associated with participation in CPRP (OR = 1.56, 95% CI: 1.12-2.16 and OR = 1.08, 95% CI: 1.01-1.15, per unit on a 5-point scale). None of the IC variables assessed could explain the large differences in health promoting behaviors between the majority and minority ethnic groups.

**Conclusions:**

IC should be taken into account in future interventions to promote physical activity and participation in CPRP for both ethnic groups. Yet, because IC failed to explain the gap between Arab and Jewish patients in those behaviors, other explanatory pathways such as psychological state or cultural views should be considered as potential areas for further research.

## Background

Cardiovascular disease (CVD) is a leading cause of death worldwide [1]. In Israel, heart disease is the second most common cause of death among the general population, and the main cause among people aged 75 or older [2].

In order to prevent recurrence after an acute coronary event, patients are advised to change lifestyle habits that are major risk factors for CVD. Specifically, there are compelling recommendations to adopt an active lifestyle [3,4]. Despite this, there continue to be large segments of the patient population that do not exercise on their own volition nor avail themselves of organized rehabilitation services. There is also an increasing awareness that ethnic minorities figure prominently among those population groups that do not adhere to these recommendations [5,6].

Participation in Cardiac Prevention and Rehabilitation programs (CPRP) is probably the most effective way to promote engagement in an active life routine. Indeed, CPRP is recommended by the major international guidelines following an acute coronary event [7,8]. Despite these recommendations, once again minorities are known to participate less in CPRP worldwide [9-11]. We have shown substantial differences between the Jewish majority and the Arab minority in Israel with regards to participation in CPRP, 61.1% and 17.2%, respectively [12].

An examination of patient-related barriers is essential to promote adoption of an active lifestyle either independently or through CPRP participation. Theories of health behavior emphasize that the way people *perceive* the situation they encounter is a crucial determinant of health-promoting behavior [13]. It is therefore plausible to assume that the challenge of equalizing services in minority ethnic groups also depends on understanding patients’ health and illness cognitions, i.e. attitudes and perceptions of their illness and the ways to cope with it. This report, theoretically based on the Health Belief Model (HBM) and the Common Sense Model (CSM), focuses on the individual’s illness cognitions as contributing to adoption of an active lifestyle and participation in CPRP among Jewish and Arab patients following acute coronary syndrome (ACS).

The Health Belief Model (HBM) [14], which has been extensively applied to explore the association between individuals’ cognitive beliefs and health behavior, is considered an effective tool for preventive health interventional planning [15].

The original model includes the following dimensions: *perceived susceptibility* (subject’s perception of the risk of contracting a condition); *perceived severity* (the medical, clinical and social consequences of the illness); *perceived benefits* (the subject’s estimate of the effectiveness of a given intervention); and *perceived barriers* (an estimate of possible negative consequences of a given behavior/intervention).

Aside from focusing on the illness situation per se, a substantial body of literature in the psychology arena has also focused on variables which characterize the patient. One important variable that has been shown to be associated with participation in CPRP is belief that the illness could be controlled [16]. This variable is particularly relevant to the current study since it focuses on the illness cognitions of two different ethnic groups, which may differ in their perceptions regarding control over the disease [17]. The current study followed Levental’s well-known common sense model (CSM) and focused specifically on the contribution of both personal control and treatment control to self-adaptive physical activity or participation in CPRP after ACS [18,19]. Personal control evaluates one’s subjective assessment of his/her ability to deal with the illness whereas treatment control taps one’s intuitive understanding of the efficacy of one’s treatment.

Although many studies have demonstrated the important contribution of illness cognition (IC) to the adoption of an active lifestyle as well as participation in CPRP [16,20-25], exploration of these associations in minority populations has been limited [26-28].

Therefore, the current longitudinal prospective study examined the contribution of illness cognition (the four HBM components together with the two CSM aspects of perceived control) to the adoption of an active lifestyle and participation in CPRP among Jewish (majority) and Arab (minority) patients hospitalized with acute coronary syndrome in an Israeli community hospital.

## Methods

The methods of the present study have been previously reported [12,29] and are summarized below.

### Study population

All patients residing in the catchment area of the Meir Medical Center in Israel admitted to the coronary care unit (CCU) whether for acute myocardial infarct (MI) or for intervention for acute coronary syndrome (ACS) between January 2009 and August 2010 were registered for the study. Distinction between MI and ACS was based on: typical history, positive EKG changes, positive troponin levels, and adjudication by a senior cardiologist. Exclusion criteria were: 1. Severe physical or mental disability that prevent participation in physical activities, 2. Inability to commit to the study (non-permanent resident in Israel, prisoners, homeless, drug addicts or alcoholics) 3. Previous participation in CPRP, 4. Lack of adequacy in Hebrew or Arabic, and 5. Immediate transfer to another institution for further treatment.

There were 649 eligible patients of whom 501 consented to be interviewed at baseline (77.2%). At six months follow up 420 consented to be interviewed, 43 refused follow up interviews, 5 had died, 26 could not be located, and 7 could not be interviewed due to their emotional/physical state yielding an 83.8% response. Response rates were similar between Arabs and Jews. There were higher response rates at follow up among men versus women, among patients admitted directly to the CCU versus transfers for interventional treatment, and among MI versus ACS patients.

The final study sample included 304 Jews (72.4%) and 116 Arabs (27.6%) with mean age of 59.6 ± 10.9 years, 84.5% were male, and 71.7% had a discharge diagnosis of acute myocardial infarction (compared to 28.3% with unstable angina).

Informed consent was obtained from each patient. The study was authorized by the Meir Hospital Institutional Review Board.

### Data collection and variable definition

Patients were interviewed face-to-face (by interviewers proficient in Hebrew and Arabic) as soon as they were stable (2–5 days after admission) regarding their socio-demographic characteristics and medical information, exercise habits, and illness cognition as described below. Information regarding participation in CPRP was gathered by telephone 6 months after discharge.

*Socio-demographic characteristics* included ethnicity (Jews/Arabs), gender, age, place of birth, marital status, education level (highest certificate/degree earned), employment status, economic situation, subjective socioeconomic position (SEP) [30], religiosity, and HMO membership. The SEP has been used previously in studies with ethnically diverse samples [31,32].

*Medical information* included the hospitalization unit (CCU or Internal medicine), diagnosis (MI or unstable angina), and history of CHD (yes/no).

Leisure-time physical activity was estimated at baseline and at follow up based on a modification of the Minnesota leisure-time physical activity (LTPA) quantitative questionnaire, which estimates activity level [33]. In this present study participants were defined as sedentary if there was no self-reported level of activity or as active if engaged in any LTPA.

To assess *participation in CPRP*, patients were asked at the follow-up interview whether they had joined any CPRP (defined as rehabilitation and not convalescence) after the index hospitalization.

Illness cognition included the HBM components and cure/control components (personal control and treatment control). The *HBM components* were measured using a questionnaire developed by Mirotznik et al. [34] for explaining attendance at a supervised CHD exercise program, based in a community center. Perceived susceptibility to CHD (3 items), perceived severity of CHD (11 items), perceived benefits of exercise (9 items), and perceived costs of exercise (5 items) were measured on a five-point Likert scale from 1 (low) to 5 (high). The average score for each subscale was used. The *cure/control components* were measured by using the revised illness perception questionnaire (IPQ-R) [35]. The full questionnaire introduced five components for assessing the patients’ cognitive representations of their illness. For the purpose of this study, personal control and self-efficacy beliefs (personal control, 6 items) and beliefs in the treatment or recommended advice (treatment control, 5 items) were used. Each items was measured on a five-point Likert scale from 1 (strongly agree) to 5 (strongly disagree). The sum score for each subscale was used. A high score represents positive beliefs about the controllability.

### Data analysis

Statistical analyses were carried out using SPSS-18 software. In unadjusted analyses, chi-square or Fisher exact tests for categorical variables and the t-test for continuous variables were used to evaluate between-group differences and assess associations between variables.

A backward stepwise logistic regression procedure with three blocks of variables and with an exit significance level of p > 0.2 was performed to determine the independent associations between the illness representations and physical activity six months after discharge. The first block included ethnicity, gender, age, and physical activity at baseline. The second block included additional socio-demographic characteristics and the medical variables, and the third block included the illness cognition variables. In order to explore the independent association between the potential cognitive barriers and participation in CPRP, the backward stepwise logistic regression procedure was repeated as above without including physical activity habits.

## Results

Arab patients reported a higher rate of a sedentary/inactive lifestyle 6 months after the index event than Jewish patients (53% vs. 18.2%).

Unadjusted Analyses **(**Table 1) Pointed to significant differences between Jews and Arabs in the illness cognition components. Compared to Jewish patients, Arabs had higher perceived susceptibility to CHD, higher perceived cost of exercise, lower perceived benefits of exercise, and lower perceived personal and treatment control. As for the association between illness cognition and adoption of an active lifestyle six months after discharge, active patients reported lower perceived susceptibility than sedentary patients in both ethnic groups [average(SD): 2.97 (0.88) vs. 3.49 (0.86), p < 0.001 among Jews and 3.06 (1.03) vs. 3.50 (0.97) among Arabs, p < 0.05], higher perceived personal control among Arabs only [average(SD): 22.89 (3.62) vs. 21.32 (3.35), p < 0.05] , and a higher perceived benefit of exercise that was evident only in Jews [average (SD): 4.00 (0.72) vs. 3.69 (0.83), p < 0.05]. Concerning participation in CPRP, participants had higher perceived personal control in both ethnic groups [average (SD): 24.21(3.87) vs. 22.22 (4.54), p < 0.001 among Jews and 23.56 (3.46) vs. 21.80 (3.53) among Arabs, p < 0.05].

**Table 1:** differences between Jews and Arabs in health belief model components and in cure/control components

**Cognitive component**	**Jews**		**Arabs**		**Total**	
	**(n)**	**M ± SD**	**(n)**	**M ± SD**	**(n)**	**M ± SD**
**Perceived susceptibility to CHD**	303	3.06 ± 0.90	113	3.29 ± 1.02*	416	3.12 ± 0.93
Perceived severity of CHD	303	3.35 ± 1.02	113	3.50 ± 0.95	416	3.39 ± 0.98
**Perceived benefits of exercise**	304	3.87 + 0.78	112	3.64 ± 0.75**	416	3.81 ± 0.78
**Perceived cost of exercise**	304	2.60 ± 0.66	111	2.87 **+** 0.73***	415	2.67 ± 0.69
**Personal control**	302	23.44 ± 4.25	111	22.11 ± 3.5**	413	23.09 ± 4.11
**Treatment control**	302	21.08 ± 2.95	111	19.92 **+** 2.57***	413	20.77 ± 2.89

*p < 0.05 **p < 0.01 ***p < 0.001.

Multivariable logistic modeling (Table 2) confirmed an independent inverse association between perceived susceptibility to CHD (OR = 0.58, 95% CI: 0.42-0.80) and a positive association between perceived personal control (OR = 1.09, 95% CI: 1.02-1.17) with exercising six months after discharge. In addition the following variables were predictors: exercise habits at the index hospitalization, ethnicity (ORArab/Jews = 0.43, 95% CI: 0.23-0.80), and education (ORhigh/low = 1.42, 95% CI: 1.10-1.82). As for the predictors of participation in CPRP (Table 3), perceived benefits of regular exercise and personal control were found to be independently associated with participation in CPRP (OR = 1.56, 95% CI: 1.12-2.16 and OR = 1.08, 95% CI: 1.01-1.15, respectively, per unit on a 5-point scale), in addition to the following predictors: ethnicity (ORArab/Jews = 0.14, 95% CI: 0.07-0.27), discharge diagnosis (ORUAP/AMI = 0.38, 95% CI: 0.22-0.65), and history of IHD (ORno/yes = 0.52, 95% CI: 0.31-0.87).

**Table 2:** **Associations of socio-demographic characteristics**, **medical variables, and cognitive variables with exercise habits six months after index hospitalization assessed by backward stepwise logistic regression**^i^

**variable**	**Block-1**	**Block-2**	**Block-3**
	**Odds ratio**^**ii **^**(95% CI), P value**	**Odds ratio**^**ii **^**(95% CI), P value**	**Odds ratio**^**ii **^**(95% CI), P value**
Exercise at baseline	3.89 (2.11-7.16), P < 0.001	3.68 (1.94-6.99), P < 0.001	3.91 (1.99-7.68), P < 0.001
Ethnicity	0.29 (0.17-0.50), P < 0.001	0.39 (0.22-0.71),P = 0.002	0.43 (0.23-0.80),p = 0.007
Gender	0.50 (0.25-1.03), P = 0.059	0.48 (0.22-1.01),P = 0.054	0.55 (0.25-1.20), p = 0.134
Age	1.02 (1.00-1.05), P = 0.112	1.03 (1.00-1.06),P = 0.035	1.03 (1.00-1.06), p = 0.057
Education		1.32 (1.04-1.67),P = 0.022	1.42 (1.10-1.82), p = 0.006
Economic situation		0.75 (0.59-0.96),P = 0.020	0.81 (0.63-1.04), p = 0.093
HMO membership		1.54 (0.83-2.86),P = 0.174	1.47 (0.78-2.79), p = 0.235
Diagnosis		2.07 (1.04-4.13),P = 0.040	1.89 (0.93-3.84), p = 0.080
Hospitalization unit		0.55 (0.29-1.06),P = 0.073	0.57 (0.30-1.11), p = 0.098
History of IHD		0.59 (0.34-1.03),P = 0.065	0.75 (0.41-1.36), p = 0.340
Perceived susceptibility to CHD			0.58 (0.42-0.80), p = 0.001
Perceived severity of CHD			1.26 (0.90-1.75), p = 0.173
Perceived benefits of exercise			0.75 (0.51-1.12), p = 0.158
Personal control			1.09 (1.02-1.17), p = 0.016
	Nagelkerke R^2^ =0.23	Nagelkerke R^2^ = 0.31	Nagelkerke R^2^ = 0.36

^i^P to exit > 0.20, within each block, so that variables in earlier blocks were retained in the final model even if their P values with the introduction of subsequent blocks increased to >0.2.

^ii^Values: Exercise habits after six months: 0 = no; 1 = yes (dependent variable); **Variables included in block-1**: Ethnic group: 0 = Jews, 1 = Arabs; Gender: 0 = male, 1 = female; Age introduced as continuous variable (years); Exercise habits at index hospitalization: 0 = no; 1 = yes; **Variables included in block-2**: SEP introduced as an ordinal variable (10 point scale from 1 = the least well off to 10 = the best off); Birth place: 0 = Israel, 1 = other; Marital status: 0 = married, 1 = other; Education level introduced as an ordinal variable (5 point scale from 1 = no formal education to 5 = Academic Education); Religiosity introduced as an ordinal variable (3 point scale from 1 = Secular to 3 = Religious); Employment status: 1 = yes; 2 = no; economic situation introduced as ordinal variable (6-point scale from 1 = excellent to 6 = very bad); HMO membership: 1 = Clalit, 2 = other; Diagnosis:0 = Myocardial infarction, 1 = Unstable Angina; History of IHD: 0 = no, 1 = yes; hospitalization unit: 1 = cardiac care unit, 2 = internal medicine; **Variable included in block-3**: Perceived susceptibility to CHD, Perceived severity of CHD, Perceived benefits of exercise, Perceived cost of exercise, Personal control, Treatment control introduced as an ordinal variable (5 point scale from 1 = not at all to 5 = very much.

**Table 3:** **Associations of socio-demographic characteristics**, **medical variables, and cognitive variables with participation in CPRP six months after index hospitalization assessed by backward stepwise logistic regression**^**i**^

**variable**	**Block-1**	**Block-2**	**Block-3**
	**Odds ratio**^**ii **^**(95% CI), P value**	**Odds ratio**^**ii **^**(95% CI), P value**	**Odds ratio**^**ii**^** (95% CI), P value**
Ethnicity	0.11(0.06-0.19),p < 0.001	0.12(0.06-0.23),p < 0.001	0.14 (0.07-0.27), p < 0.001
Age	0.97 (0.95-0.99), p = 0.009	0.98(0.96-1.01),p = 0.143	0.99 (0.97-1.02),p = 0.475
SEP		1.14(1.01-1.29),p = 0.035	1.11 (0.98-1.26), p = 0.099
Marital status		0.59(0.32-1.10),p = 0.097	0.60 (0.31-1.14), p = 0.119
Education		1.22(0.99-1.49),p = 0.062	1.21 (0.98-1.49), p = 0.073
HMO membership		0.63 (0.37-1.09), p = 0.096	0.58 (0.33-1.01), p = 0.054
Diagnosis		0.33 (0.22-0.65),p < 0.001	0.38 (0.22-0.65), p < 0.001
History of IHD		0.55 (0.33-0.90), p = 0.018	0.52 (0.31-0.87), p = 0.013
Perceived benefits of exercise			1.56 (1.12-2.16), p = 0.009
Personal control			1.08 (1.01-1.15), p = 0.017
	Nagelkerke R^2^ =0.21	Nagelkerke R^2^ = 0.32	Nagelkerke R^2^ = 0.36

^i^P to exit > 0.20, within each block, so that variables in block 1 (ie. age) were retained in the final model even if their P values with the introduction of subsequent blocks increased to >0.2.

^ii^Values: Exercise habits after six months: 0 = no; 1 = yes (dependent variable); **Variables included in block-1**: Ethnic group: 0 = Jews, 1 = Arabs; Gender: 0 = male, 1 = female; Age introduced as continuous variable (years) **Variables included in block-2**: SEP introduced as an ordinal variable (10 point scale from 1 = the least well off to 10 = the best off); Birth place: 0 = Israel, 1 = other; Marital status: 0 = married, 1 = other; Education level introduced as an ordinal variable (5 point scale from 1 = no formal education to 5 = Academic Education); Religiosity introduced as an ordinal variable (3 point scale from 1 = Secular to 3 = Religious); Employment status: 1 = yes; 2 = no; economic situation introduced as ordinal variable (6-point scale from 1 = excellent to 6 = very bad); HMO membership: 1 = Clalit, 2 = other; Diagnosis:0 = Myocardial infarction, 1 = Unstable Angina; History of IHD: 0 = no, 1 = yes; hospitalization unit: 1 = cardiac care unit, 2 = internal medicine; **Variable included in block-3**: Perceived susceptibility to CHD, Perceived severity of CHD, Perceived benefits of exercise, Perceived cost of exercise, Personal control, Treatment control introduced as an ordinal variable (5 point scale from 1 = not at all to 5 = very much.

Thus, the strong contribution of ethnicity to both independent physical activity and CPRP-based physical activity persisted, notwithstanding the significant contribution of several of the illness cognitions assessed.

Addition of interaction terms of ethnicity with each of the illness cognitions yielded no significant contribution.

## Discussion

In 2012 Stuart-Shor et al. [36] published a comprehensive review of the significant impact behavioral factors have on explaining the substantial differences in cardiovascular outcomes in ethnic minorities. This review extensively discussed strategies for altering disease perceptions and made special mention of “cross-cultural” factors including the meaning of illness and wellness as being important in altering patient behavior.

The current findings demonstrated the contribution of perceived susceptibility to CHD, perceived benefit of regular exercise, and sense of personal control to adoption of an active lifestyle or participation in CPRP among Jewish and Arab ACS patients. Perceived susceptibility and sense of personal control were independently associated (inversely and positively, respectively) with an active lifestyle 6 months after the acute event. Perceived benefits of regular exercise was not found to be associated directly with an active lifestyle but was associated with participation in CPRP - a surrogate for organized physical activity. Sense of personal control was also associated with participation in CPRP.

It seems that cardiac patients who conceive themselves to be highly predisposed to additional cardiac events refrain from exercising on a regular basis. A possible explanation may be that these patients misconceive this behavior as harmful to their health. Interestingly, susceptibility was not associated with joining CPRP. Altering this perception by reassuring patients with regard to the safety and efficacy of CPRP may be an effective intervention to overcome this barrier.

In the same vein, patients who conceive exercise to be beneficial compared with patients who overlooked these benefits tend to join CPRP but not necessarily to engage in physical activity on their own. Thus, CPRP seems to be a useful scaffold for initiating an active life style for those patients who already regard this behavior as beneficial. Unfortunately, those patients who do not perceive exercise as beneficial tend to participate less in CPRP and, as a consequence, might be at higher risk for recurrence of cardiac events. Patients who perceived themselves as capable of controlling their illness were more likely to initiate an active lifestyle and to participate in CPRP. Therefore, interventions targeted at alleviating patients’ misconceptions regarding susceptibility to CHD, benefits of exercise and lack of personal control are highly recommended.

Notwithstanding the importance of these findings, none of the illness cognitions assessed in the current study was able to explain the difference in health promoting behaviors between the Jewish majority and the Arab minority in our sample. Our findings among cardiac patients are consistent with those of another recent study which also demonstrated the inability of health-related perceptual variables to explain ethnic differences in physical activity, this time within the general population [28].

The following limitations of our study should be considered. First, the response rate, although generally favorable, was lower among those transferred for intervention, among those admitted for unstable angina, and among women, especially Arab women. Second, the lower participation rates of Arab patients in CPRP, a phenomenon well recognized among ethnic minorities, affected the power to adequately explore interactions of ethnicity with illness cognition. “Third, in our analysis we incorporated a dichotomous yes/no response based on levels of reported exercise at follow up, while controlling for baseline level activity. We deliberately refrained from predicting actual levels of physical activity (energy expenditure), as the focus of the current publication was the stage of change in which an actual action is taken and the person is acquiring a new health behavior, in this instance from sedentary to active lifestyle”. An additional limitation is the lack of data regarding length of participation in CPRP. Estimates were made at the follow up interview and the minimum period to count as participation was one month. Actual lengths were quite variable including many patients who continue ongoing participation as part of their life style change. Finally, the current study did not distinguish between Jewish immigrants and veteran Israelis although lower rates of participation in CPRP have been demonstrated among immigrants [37]. All these limitations should be taken into account in future research.

## Conclusions

IC should be taken into account in future interventions to promote physical activity and participation in CPRP for both ethnic groups. The finding that the strong ethnic difference in exercise habits and in participation in CPRP still persists beyond the psychosocial determinants and the illness representations, suggests the existence of other explanatory pathways such as psychological state or cultural world views. Further studies are needed in order to elucidate this issue for the benefit of both populations.

## Competing interests

The authors declare that they have no competing interests.

## Authors’ contributions

OR participated in the creation of the study’s concept and design, organized and took part in the data collection, carried out statistical analysis, took part in the interpretation of the data, and drafted the manuscript. NV participated in the creation of the study’s concept and design, provided advice and guidance on statistical and psychological issues, took part in the interpretation of the data, and critically revised the draft. ML participated in the creation of the study’s concept and design, supervised the data collection from a medical perspective, took part in the interpretation of the data, critically revised the draft, and helped obtain funding. AK contributed to the conception of the study, provided medical advice during the data acquisition/collection phase, and helped in the design of the questionnaires. MM took part in the interpretation of the data, critically revised the draft, supervised the study, and obtained funding. JDK contributed to the study’s conception and design, advised on statistical and epidemiological issues, took part in the interpretation of the data, and critically revised the draft. All authors read and approved the final manuscript.

## Pre-publication history

The pre-publication history for this paper can be accessed here:

http://www.biomedcentral.com/1471-2458/13/956/prepub
